# Neurotransmitters in Auditory Processing Disorders and Neurodevelopmental Disorders: A Common Neurobiological Substrate?

**DOI:** 10.3390/children13050697

**Published:** 2026-05-19

**Authors:** Andrea Bianchino, Andrea Migliorelli, Marianna Manuelli, Chiara Bianchini, Francesco Stomeo, Stefano Pelucchi, Andrea Ciorba, Luca Sacchetto, Silvia Palma, Daniele Monzani

**Affiliations:** 1 ENT and Audiology Department, University Hospital of Ferrara, 44124 Ferrara, Italy; andrea.bianchino@edu.unife.it (A.B.); mglndr1@unife.it (A.M.); marianna.manuelli@ospfe.it (M.M.); chiara.bianchini@unife.it (C.B.); francesco.stomeo@unife.it (F.S.); stefano.pelucchi@unife.it (S.P.); andrea.ciorba@unife.it (A.C.); 2 ENT and Audiology Department, University Hospital of Verona, 37126 Verona, Italy; luca.sacchetto@univr.it; 3 Audiology, Primary Care Department, Ausl Modena, 41121 Modena, Italy

**Keywords:** auditory processing disorder, neurodevelopmental disorders, central auditory processing, GABAergic inhibition, glutamatergic transmission, dopaminergic system, serotonergic system, excitation–inhibition balance

## Abstract

**Highlights:**

**What are the main findings?**

- Auditory processing difficulties may represent sensory-level expressions of shared neurochemical vulnerability across neurodevelopmental conditions.
- The widespread co-occurrence of auditory processing difficulties within neurodevelopment disorder populations indicates that a deeper understanding of auditory dysfunction may help clarify underlying mechanisms across the neurodevelopmental spectrum.

**What are the implications of the main findings?**

- Recognizing the link between NDDs and auditory processing disorders carries important clinical implications for early identification, differential diagnosis, and the design of targeted interventions.
- Future research should incorporate concurrent measurements of multiple neurotransmitter systems within auditory-related brain regions to better characterize disorder-specific patterns of dysfunction.

**Abstract:**

**Background/Objectives**: Auditory processing disorders (APDs), defined as impaired neural processing of acoustic stimuli despite normal peripheral hearing, often co-occur with neurodevelopmental disorders (NDDs) and may contribute to language, attentional, and learning difficulties. Emerging evidence suggests that shared neurotransmitter systems may represent a common neurobiological substrate underlying these conditions. The aim of this study is to integrate current evidence on glutamatergic, GABAergic, and monoaminergic systems in neurodevelopmental and auditory processing disorders in children and adolescents, and to evaluate the hypothesis that shared neurotransmitter dysregulation may underlie their clinical overlap. **Methods**: A narrative review of the literature was conducted through electronic searches in PubMed and Embase up to 31 December 2025, using keywords related to neurotransmitters, NDDs and APDs. **Results**: Available evidence indicates that an imbalance between excitatory glutamatergic and inhibitory GABAergic neurotransmission has been proposed as a central mechanism in NDDs and may also contribute to auditory processing difficulties through altered neural synchrony, sensory gating and temporal auditory coding. Findings collectively suggest the hypothesis of shared neurotransmitter dysregulation across NDDs and APDs. **Conclusions**: Auditory processing difficulties may represent sensory-level expressions of shared neurochemical vulnerability across neurodevelopmental conditions. Future longitudinal and multimodal studies are needed to clarify causal relationships and to identify clinically useful biomarkers.

## 1. Introduction

Auditory processing disorders (APDs), including the so-called central auditory processing disorder (CAPD), refer to difficulties in the neural analysis of acoustic stimuli despite normal peripheral hearing thresholds [1,2]. Typical manifestations include reduced speech understanding in noise, temporal processing deficits, and broader listening difficulties in acoustically complex environments [3]. APD is characterized by impaired sound recognition and sound-source localization, reflecting functional alterations of the central auditory system [1,2], and frequently co-occurs with dyslexia, developmental language disorders, autism spectrum disorder (ASD) and attention-deficit/hyperactivity disorder (ADHD) [2]. These last two disorders are highly prevalent and frequently comorbid conditions [4,5].

Neurodevelopmental disorders (NDDs) encompass a heterogeneous group of conditions characterized by atypical brain development that, to varying degrees, affect cognition, behavior, and socio-communicative functioning [6,7]. Although APD and NDDs have traditionally been conceptualized as distinct entities, increasing evidence indicates that auditory processing alterations are common across several neurodevelopmental conditions and have been associated with language, attentional, and learning difficulties [6,8]. Over the past decade, neurobiological research has highlighted potentially convergent mechanisms across different NDDs, with particular attention to the balance between excitatory and inhibitory synaptic transmission [7,9]. According to this hypothesis, disruptions in glutamatergic excitatory transmission and GABAergic inhibitory signaling may destabilize neural circuits and interfere with experience-dependent plasticity [10,11]. These alterations have been linked to clinical features such as sensory hyper/hypo-reactivity, attentional difficulties, repetitive behaviors, and executive dysfunction [5,6].

Auditory perception, in particular, relies on highly precise temporal regulation of the excitation/inhibition (E/I) balance throughout the brainstem to cortical regions, to support rapid temporal coding, signal extraction from noise, and sensory gating mechanisms [12,13]. Beyond glutamatergic and GABAergic transmission, monoaminergic systems play a critical modulatory role in arousal regulation, attentional control, reward processing, and sensory gain modulation [5,14]. These neurotransmitters influence neuronal responsiveness, shape synaptic plasticity, and coordinate higher-order functional networks involved in executive control and selective attention [15]. Monoaminergic dysregulation is well documented in ADHD and has also been described in ASD and other NDDs [16,17]. At the same time, growing evidence suggests that these systems contribute to central auditory function, particularly in attentional selection, sensory filtering, and temporal precision [18,19]. This convergence raises the possibility that, in a subset of individuals, listening difficulties may reflect a shared neurochemical vulnerability rather than an “isolated auditory” deficit.

Despite these premises, the relationship between neurotransmitter systems and auditory processing disorder remains insufficiently systematized. Evidence is scattered across different disciplines, including audiology and neuroscience, and derives from heterogeneous methodologies such as behavioral testing, electrophysiology, magnetic resonance spectroscopy (MRS), pharmacological studies, and translational animal models [10,20,21]. Clarifying whether plausible shared mechanisms exist, and to what extent, carries important clinical implications for early identification, differential diagnosis, phenotypic stratification, and the development of more targeted interventions [8,22]. The aim of this study is to synthesize the literature on the role of major neurotransmitter systems (glutamatergic, GABAergic and monoaminergic) in neurodevelopmental and APD and to evaluate the hypothesis of a neurobiological link between NDDs and APD.

## 2. Materials and Methods

### Search Sources and Strategy

A literature search was conducted using the PubMed and Embase databases to identify English-language studies addressing the role of neurotransmitters in NDDs and APDs. The following keywords were used: (“neurotransmitter*” OR “neurotransmission” OR GABA OR “gamma-aminobutyric acid” OR glutamate OR Glx OR dopamine OR serotonin OR 5-HT OR norepinephrine OR noradrenaline) AND (“neurodevelopmental disorder*” OR NDD OR autism OR “autism spectrum disorder” OR ASD OR ADHD OR “attention-deficit/hyperactivity disorder” OR “tuberous sclerosis complex” OR “fragile X syndrome”) AND (“auditory processing disorder” OR APD OR “central auditory processing disorder” OR CAPD OR “auditory processing” OR “listening difficult*” OR “speech in noise”). The electronic search was complemented by manual screening of the reference lists of selected articles to identify additional relevant publications. The literature search included studies published from 2006 up to 31 December 2025.

Articles published in English in peer-reviewed scientific journals, including both original studies and literature reviews, relevant to the role of neurotransmitter systems in neurodevelopmental disorders and central auditory processing disorders, were included. Studies conducted on both human populations and animal models were considered, provided they were relevant to understanding the neurobiological mechanisms of auditory processing. Book chapters, conference proceedings, editorials, letters, case reports, and works not relevant to the topic of neurotransmission in neurodevelopmental or auditory processing disorders were excluded. Studies that focused exclusively on peripheral hearing loss without reference to central auditory processing mechanisms were also excluded. Given the narrative nature of this review, a formal assessment of the methodological quality of the included studies was not performed; however, priority was given to studies with clear methodological descriptions and direct relevance to the neurobiological mechanisms under analysis. For the same reason, a formal systematic selection process with predefined record counts was not performed. Studies were selected based on their relevance to the research question and methodological clarity. The screening and selection process was conducted by two authors to ensure consistency in study inclusion. Discrepancies in the selection process were resolved, when necessary, by consulting a senior reviewer.

## 3. Results

### 3.1. Neurodevelopmental Disorders

#### 3.1.1. Role of GABA and Glutamate

Increasing evidence indicates that reduced inhibitory GABAergic transmission together with enhanced excitatory glutamatergic signaling has been associated with alterations in cognitive and memory development [7,23,24]. Disruption of this balance has been reported as a central pathophysiological mechanism across several NDDs, including ASD and ADHD [9].

A comparative overview of GABAergic and glutamatergic alterations across major neurodevelopmental and psychiatric conditions is provided in Table 1.

The E/I imbalance has been reported as a shared feature of ASD and ADHD and is associated with phenotypic heterogeneity including repetitive behaviors, attentional deficits, and learning difficulties [23]. In NDDs, particularly ASD, individuals frequently exhibit altered gene expression related to GABAergic signaling along with disrupted glutamate homeostasis [9]. Reduced GABAergic tone has been hypothesized to contribute to cortical hyperexcitability, whereas elevated glutamate levels may promote excitotoxic processes through excessive calcium influx and mitochondrial dysfunction [25]. Metabolomic studies further reveal disturbances in glutamate and GABA metabolism in both plasma and cerebrospinal fluid among individuals with NDDs [26]. Alterations in amino-acid profiles in children with ADHD, including changes in glutamatergic pathways and in the glutamate–glutamine ratio, have also been reported [27]. Developmental trajectories of brain GABA and glutamate concentrations appear non-linear across childhood and adolescence, reflecting neurodevelopmental processes such as synaptic refinement and circuit specialization [28]. For instance, children with ASD have been reported to show initially lower parietal GABA+ levels compared with typically developing peers, with normalization occurring during early adolescence and gradual increases thereafter [29]. These findings highlight the dynamic and region-specific nature of E/I balance during neurodevelopment. In contrast, glutamate/glutamine (Glx) concentrations exhibit distinct developmental patterns that may contribute to the heterogeneity of symptom expression across the lifespan. Reduced GABA concentrations in the sensorimotor cortex and related motor areas correlate with specific clinical phenotypes [30]. Lower GABA levels in the left ventral premotor cortex, for example, have been associated with heightened sensory reactivity, indicating impaired modulation of incoming stimuli. Similarly, reduced thalamic GABA concentrations have been linked to greater sensory sensitivity [30]. Conversely, elevated GABA levels have been reported in the dorsolateral prefrontal cortex of adults with ASD [31]. Glutamatergic abnormalities in ASD are similarly complex. Findings include reduced striatal glutamate associated with greater severity of social symptoms [32,33] as well as elevated plasma glutamate levels [34]. In ADHD, neurochemical studies indicate reduced activation of both glutamate and GABA within fronto-striatal circuits during cognitively demanding tasks compared with neurotypical controls [35]. Alterations in GABAergic regulation contribute to the neurophysiological abnormalities of auditory sensory processing observed in ASD [36]. Children with ASD often exhibit hypersensitivity to higher-frequency tones and a broader temporal integration window compared with typically developing peers, factors that may contribute to sensory overload and difficulties in processing auditory input. Prolonged latencies in click-evoked auditory brainstem responses (click-ABR) have also been reported in this population, suggesting alterations in sensory gating mechanisms and audiovisual temporal integration, both considered characteristic features in ASD [29,30,31,32,33,34,35,37].

#### 3.1.2. Role of Monoamines

Dopamine, serotonin, and norepinephrine are monoaminergic neurotransmitters involved in motor regulation, reward processing, mood modulation, attention, and broader cognitive functions essential for typical neurodevelopment [38]. The main monoaminergic neurotransmitters and their principal neurobiological functions, together with their potential relevance to neurodevelopmental and auditory processing disorders, are summarized in Table 2. Dysregulation of these systems has emerged as a central mechanism across multiple NDDs, with alterations manifesting at different developmental stages and within distinct neural circuits [39]. Clinical heterogeneity of NDDs indicates the involvement of multiple biological mechanisms, among which monoaminergic dysfunction remains one of the most consistently observed.

Serotonin has been extensively studied in NDDs, particularly in ADHD, where alterations in synthesis, transport, and degradation have been reported. Most studies indicate broad involvement of the serotonergic system in ADHD [16]. In ASD, around 30% of individuals exhibit elevated blood serotonin levels. The modulatory role of serotonin supports its involvement in neurodevelopmental processes and in the clinical expression of autism-related traits [17]. Beyond mood regulation, serotonin has been reported to influence sensory responsiveness, behavioral reactivity, and a range of neuropsychological processes in NDDs. Epigenetic polymorphisms of the TPH2 gene—encoding a key enzyme in serotonin synthesis—have been associated with the severity of attentional and impulsive symptoms, suggesting a potential role as a predictive biomarker [17].

### 3.2. Auditory Processing Disorder

#### 3.2.1. Role of GABAergic and Glutamatergic Systems

Central auditory processing relies on complex synaptic interactions within brainstem, midbrain, and cortical circuits, where GABAergic inhibition and glutamatergic excitation operate in a tightly coordinated manner [12]. The central auditory system is organized as a hierarchical network of circuits that progressively transform acoustic information into perceptual representations [39,40,42,43]. APD reflects a dysfunction in how the central nervous system analyzes and organizes acoustic information. As synaptic plasticity allows synapses to adjust their strength in response to changing patterns of neural activity [10,43,44], disturbances in neurotransmitter homeostasis can substantially impair auditory circuit function. The principal neurotransmitter systems implicated in APDs and their proposed functional roles are summarized in Table 3. GABAergic inhibition plays an important role in shaping the temporal response properties of auditory neurons, refining frequency selectivity and suppressing background noise. Glutamatergic transmission provides the primary excitatory drive, thereby facilitating both signal propagation and adaptive synaptic refinement. Tonotopic organization of the auditory system requires precise coordination of glutamatergic and a GABAergic presynaptic maturation processes [45].

A pivotal study employing magnetic resonance spectroscopy to quantify neurotransmitter concentrations demonstrated that patients with tinnitus, despite having normal hearing thresholds, exhibited reduced glutamate levels in the right primary auditory cortex and decreased GABA concentrations in the left primary auditory cortex [20]; glutamate levels showed a positive correlation with tinnitus loudness ratings, suggesting a possible link between neurochemical profile and “phantasm” symptom severity [20]. A meta-analysis integrating proton magnetic resonance spectroscopy with functional MRI demonstrated an inverse association between regional GABA concentration and task-related neural activation, both in visual cortex and in prefrontal regions involved in emotional regulation [46]. GABAergic inhibition appears to actively shape the spatial and temporal dynamics of neuronal population responses. Alterations in GABAergic tone have been associated with changes in the temporal and spectral dynamics involved in speech perception, auditory scene analysis, and the integration of acoustic information with higher cognitive functions. The 40 Hz auditory steady-state response (ASSR) represents a neurophysiological marker of the ability of neuronal populations to synchronize with rhythmic auditory stimulation [11]. In the human auditory cortex, the 40 Hz auditory steady-state response has been reported to depend primarily on GABAergic synaptic inhibition rather than glutamatergic excitation [12]. Deficits in GABAergic signaling have been associated with impaired neural synchrony required for accurate temporal coding and signal-to-noise discrimination in complex auditory environments. Alterations in resting-state brain oscillations observed on electroencephalography (EEG) in children with CAPD suggest an imbalance between E/I mechanisms affecting both auditory-processing regions and broader supporting neural networks. Specifically, children with APD [47] showed an electrophysiological profile that decreased functional engagement of cortical regions involved in auditory processing and attentional control. Such a pattern has been interpreted as reflecting reduced GABA-mediated inhibitory regulation. The relationship between resting-state frequency bands and performance on CAP tests has been reported, and these oscillatory alterations have been proposed as potential neurophysiological biomarkers of listening difficulties in APD. Atypical development or dysfunction of inhibitory circuits has been associated with impairments in auditory perception. Key neurophysiological and neurochemical markers linked to E–I imbalance in auditory processing disorders are summarized in Table 4.

Studies on long-latency auditory evoked responses indicate that children with central APD often exhibit slower cortical responses and reduced amplitudes compared with peers who have normal hearing [48]. Among these components, N1 and N2 appear particularly informative in distinguishing children with APD from controls, suggesting their potential role as biological markers of the disorder.

The inhibitory tone exerted by pyramidal neurons also plays a relevant role in regulating neuronal activation thresholds and mismatch negativity (MMN), an important electrophysiological marker of auditory change detection. Experimental and computational studies suggest that alterations in pyramidal cell self-inhibition can modulate neuronal excitability and are associated with reduced MMN amplitude, supporting a link between inhibitory control mechanisms and auditory predictive processing [49].

Children with CAPD also tend to show more pronounced differences in sensory processing across multiple modalities compared with typically developing peers, including visual, tactile, and proprioceptive systems [50]. This could indicate a systemic dysfunction of E/I balance affecting multisensory neural networks. Prenatal and early postnatal exposure to stressors may also lead to persistent alterations in the balance between GABAergic and glutamatergic neurotransmission [51].

Concerning the development of the central auditory system, evidence suggests that during specific stages of gestation, nutrients such as choline and folic acid can influence the timing of neurodevelopmental processes. In particular, cholinergic signaling appears to facilitate the developmental switch of GABA receptor function from excitatory to inhibitory, as well as the recruitment of GluR1–R2 glutamate receptor subunits that support faster glutamatergic responses [52]. The maturation of inhibitory signaling has been associated specifically with maternal plasma choline levels and folic acid supplementation around the 16th week of gestation, whereas the development of excitatory signaling appears primarily related to maternal choline levels at approximately 28 weeks [52]. Alterations in E–I balance are closely linked to the activity of ion-channel subunits, and mutations affecting these channels may contribute to both central auditory and cognitive deficits [53]. Variants affecting this channel may alter neuronal excitability thresholds by modifying Ca^2+^ influx, thereby influencing excitatory–inhibitory tone and contributing to emotional and behavioral deregulations [54].

#### 3.2.2. Role of Monoamines

Monoaminergic systems appear to modulate auditory processing at multiple hierarchical levels, from basic sensory gain control to higher-order attentional and cognitive integration [40]. They contribute to auditory processing by linking acoustic input with attention, motor responses, and the motivational salience of stimuli. Monoaminergic projections—particularly serotonergic and dopaminergic pathways—play an important role in central auditory processing at both cortical and subcortical levels. Serotonin, in particular, densely innervates the primary auditory cortex as well as thalamic nuclei projecting to cortical auditory regions, supporting its potential as a pharmacological target in APD [15]. By contrast, dopaminergic involvement in auditory processing appears more circumscribed and largely related to attentional modulation.

Auditory processing difficulties show considerable heterogeneity across clinical conditions, and the overlap between APD and ADHD likely reflects partially distinct monoaminergic pathophysiologies [55]. While ADHD is primarily characterized by dysregulation of dopaminergic and noradrenergic systems that support executive attention and response inhibition [41], APD may more specifically involve serotonergic and dopaminergic dysfunction within auditory-related neural circuits [18,19].

The main functional roles and auditory-related implications of dopamine, serotonin, and norepinephrine are summarized in Table 5.

Dopamine plays a critical role in reward processing and motor control [56], acting through distinct neural circuits that influence motivated behavior as well as motor function. Both iontophoretic application and optogenetically induced dopamine release have been shown to modulate inferior colliculus neuronal responses, with dopamine capable of either enhancing or suppressing neuronal activity depending on cellular context [18]. Additionally, dopamine acts by increasing the intrinsic excitability of fast-spiking parvalbumin-expressing GABAergic interneurons [57], a mechanism that alters the balance between excitation and inhibition in sensory cortices. Parvalbumin-expressing interneurons are critical for the generation of oscillatory activity and for the precise temporal coordination of pyramidal neuron firing, making them essential for accurate temporal encoding of auditory information. Altered dopaminergic tone has been associated with changes in neural filtering, selective attention to auditory stimuli, and suppression of background noise, as well as with auditory processing deficits in disorders characterized by dopaminergic dysregulation.

Dopaminergic signaling also modulates the transfer of auditory information toward higher-order processing regions, with dopamine-sensitive corticocortical pathways influencing the processing of emotionally salient auditory stimuli within the orbitofrontal cortex [58]. Pharmacological dopaminergic agonists reduce acoustic responses in the orbitofrontal cortex with longer action kinetics compared with primary auditory areas, suggesting layer-specific and region-specific integration of dopaminergic modulation within hierarchical auditory networks.

Serotonin exerts various effects on auditory processing through distinct receptor mechanisms and transporter-dependent signaling, influencing temporal processing, multisensory integration, and central auditory function. It is involved in mood regulation and sensory processing [14]. At the level of temporal encoding, serotonin acts via 5-HT1A receptors to normalize auditory temporal processing deficits in developmental models. Acute administration of the selective 5-HT1A agonist NLX-101 has been shown to significantly reduce gamma power while increasing inter-trial phase clustering in auditory responses [19]. This pharmacological intervention improves temporal phase locking without altering event-related potential amplitudes, suggesting that serotonergic modulation targets specific neural mechanisms underlying precise acoustic timing independently of classical evoked auditory responses. Such dissociable effects point toward layer-specific or circuit-specific serotonergic actions that preserve certain aspects of auditory coding while enhancing the temporal fidelity required for speech and language development. Serotonergic dysfunction seems to influence both peripheral and central auditory responses. Treatment with selective serotonin reuptake inhibitors has been shown to reversibly improve auditory processing deficits and abnormal screening outcomes in central auditory processing [19]. Pharmacologically treated patients demonstrated normalized performance on central auditory processing tests together with significantly improved auditory brainstem responses compared with untreated conditions.

Norepinephrine contributes to the coordinated modulation of primary afferent neurotransmission through presynaptic inhibitory mechanisms. Experimental evidence indicates that noradrenaline modulates dorsal root potentials and monosynaptic field responses in the dorsal horn via metabotropic receptor activation, producing depressive effects comparable to dopamine but generally weaker than those observed with serotonin [40,41,55,56,57,58,59]. Beyond the modulation of spinal afferents, noradrenaline plays a central role in the attention regulation of auditory processing through its involvement in the brain’s arousal neuromodulatory system [14]. Acting in concert with dopamine and acetylcholine to shape distinct behavioral states, noradrenaline provides the flexibility and stability required for adaptive behavior, including the dynamic allocation of attentional resources to incoming auditory information. Deregulation of noradrenergic signaling may therefore compromise arousal and vigilance mechanisms that are essential for sustaining attention in acoustically challenging environments. The functional distinction between noradrenaline’s role in arousal regulation and dopamine’s involvement in reward processing and motor control further suggests that different monoaminergic systems may preferentially support specific aspects of auditory function. Within this framework, noradrenaline appears to provide the attentional substrate upon which accurate auditory processing depends, facilitating the selection, prioritization, and stabilization of behaviorally relevant acoustic signals.

#### 3.2.3. Acetylcholine

Research indicates a significant link between the cholinergic system, auditory processing, and ASD. Acetylcholine (ACh) has a role in attention, novelty seeking, and memory. ACh deficits, particularly in nicotinic acetylcholine receptors (nAChR), are associated with auditory processing differences, including hypersensitivity, delayed ABRs, and impaired auditory temporal processing in autistic individuals [60,61].

nAChRs are essential for auditory processing and are present in the auditory brainstem. Nicotinic receptors are expressed postsynaptically before hearing onset and switch to presynaptic expression after hearing onset [62]. On the other side, evidence concerning the effectiveness of acetylcholinesterase inhibitors as a medication to improve outcomes for autistic children is very uncertain [63]. Concerning the nicotinic acetylcholine receptor subunits alpha9 and alpha10, they have been identified as critical components of the efferent auditory system and medial olivocochlear pathway. This pathway has been reported to modulate outer hair cell function and signal detection in noise [64].

The gene encoding the α7 subunit of the nicotinic acetylcholine receptor (α7-nAChR) has been associated with ASD. In this context, auditory brainstem response (ABR) studies have shown that loss of α7-nAChR function is associated with abnormal auditory temporal processing [65].

Moreover, emerging evidence suggests that personalized interventions targeting specific monoaminergic receptor profiles [58] may offer therapeutic benefits for selected patient subgroups with auditory processing deficits linked to underlying monoaminergic dysregulation. The implementation of these precision neuropharmacology strategies will require the development of reliable biomarkers capable of identifying individual patterns of monoaminergic dysfunction, enabling stratified clinical trial designs in which treatments are matched to neurochemical profiles rather than uniformly applied across heterogeneous patient populations.

## 4. Conclusions

The widespread co-occurrence of auditory processing difficulties within NDD populations indicates that a deeper understanding of auditory dysfunction may help clarify shared underlying mechanisms across the neurodevelopmental spectrum. Recognizing the link between NDDs and APD carries important clinical implications for early identification, differential diagnosis, and the design of targeted interventions [21]. Current evidence suggests that developmental alterations in sensory signal processing during critical neurodevelopmental windows may lead to persistent modifications in the neural circuitry underlying auditory responsiveness and processing. Because developing auditory circuits rely critically on balanced excitatory–inhibitory activity to establish functional organization and stable connectivity, early disruptions of this balance may have long-lasting consequences for the integrity of the central auditory system. This highlights the importance of early interventions specifically addressing sensory processing dysfunction. Within this framework, APD and NDDs are increasingly viewed not as entirely independent diagnostic entities, but rather as potentially interconnected manifestations of altered neurodevelopmental trajectories.

The boundaries between auditory processing disorders and other neurodevelopmental conditions remain unclear, with substantial clinical and behavioral overlap complicating differential diagnosis and suggesting shared underlying mechanisms. An imbalance between E/I has emerged as a unifying mechanism that may account for auditory dysfunction across multiple neurodevelopmental conditions [6]. Disruption of synaptic homeostasis can create a neural environment in which excitatory and inhibitory influences are poorly regulated, producing cascading effects throughout auditory circuits. The co-occurrence of sensory and cognitive deficits in ASD, ADHD, APD, and other neurodevelopmental conditions appears to be shaped by specific neurochemical patterns as well as by the presence of comorbidities [8]. Given the substantial overlap in behavioral symptoms between neurodevelopmental disorders and APD, accurate diagnosis often requires a multidisciplinary approach to improve both diagnostic precision and the personalization of therapeutic strategies [8].

Future research should incorporate concurrent measurements of multiple neurotransmitter systems within auditory-related brain regions to better characterize disorder-specific patterns of dysfunction [66]. Such multi-neurotransmitter profiling could help identify distinct monoaminergic signatures associated with specific auditory phenotypes, moving beyond current single-neurotransmitter diagnostic and therapeutic approaches.

## Figures and Tables

**Table 1:** Both neurodevelopmental and psychiatric conditions show convergent alterations in E-I, although regional distribution and clinical manifestations differ substantially across disorders.

Disorder	GABAergic Alterations	Glutamatergic Alterations	Principal Regions Implicated	Clinical Correlates
Autism spectrum disorder (ASD)	Reduced parietal GABA+ in childhood; decreased sensorimotor and thalamic GABA; increased dorsolateral prefrontal GABA in adults	Reduced striatal glutamate; elevated plasma glutamate; altered Glx developmental trajectories	Sensorimotor cortex; ventral premotor cortex; thalamus; striatum; dorsolateral prefrontal cortex	Sensory hyper-reactivity; social impairment; cortical hyperexcitability
Attention-deficit/hyperactivity disorder (ADHD)	Reduced GABAergic activation during inhibitory control tasks	Reduced glutamatergic activation in fronto-striatal circuits	Prefrontal cortex; fronto-striatal networks	Inattention; impulsivity; impaired response inhibition
Schizophrenia [7]	Reduced cortical GABAergic inhibition	Hyperglutamatergic states; altered glutamate/GABA ratio	Prefrontal cortex; widespread cortical networks	Psychosis; working memory deficits; executive dysfunction

**Table 2:** Main monoaminergic neurotransmitters and their principal neurobiological functions, together with their potential relevance to neurodevelopmental and auditory processing disorders.

Neurotransmitter	Core Neurobiological Functions	Evidence in NDDs	Potential Implications for Auditory Processing
Dopamine [38,39]	Reward processing, motor control, motivation, executive function, attentional modulation	Altered dopaminergic signaling consistently reported in ADHD and ASD; linked to attentional deficits, impulsivity, and altered behavioral learning	May influence auditory attention, salience attribution, and top-down modulation of auditory cortical responses
Serotonin [16,17]	Mood regulation, sensory modulation, emotional reactivity, neurodevelopmental plasticity	Hyperserotoninemia observed in a subset of ASD; altered serotonin synthesis, transport, and receptor expression in ADHD	Implicated in sensory gating, temporal auditory processing, and modulation of sensory responsiveness
Noradrenaline [40,41]	Arousal regulation, vigilance, attentional control, stress responsivity	Dysregulation associated particularly with ADHD and attentional control disorders	May modulate auditory signal-to-noise processing, listening effort, and sustained auditory attention

**Table 3:** Principal neurotransmitter systems implicated in APDs and their proposed functional roles.

Neurotransmitter System	Physiological Role in Auditory Processing	Evidence in APD and Related Conditions	Clinical Implications
GABAergic inhibition [11,12,20]	Regulates temporal precision, sharpens frequency tuning, suppresses background noise, supports sensory gating and gamma oscillations	Altered cortical GABA levels (MRS); reduced auditory repetition suppression; ASSR sensitivity to GABAergic modulation	Potential biomarker of auditory filtering deficits; target for GABA-B receptor modulation
Glutamatergic excitation [20,33]	Provides primary excitatory drive; supports signal propagation and activity-dependent synaptic plasticity	Reduced or regionally altered glutamate/Glx levels in tinnitus and ASD; association with perceptual deficits	Possible role in speech-in-noise impairment and maladaptive plasticity
Excitation–inhibition (E/I) balance [21,46]	Optimizes signal-to-noise ratio; maintains tonotopic organization; enables precise temporal coding	Animal models (e.g., Cntnap2); electrophysiological and MRS evidence of imbalance	Conceptual framework linking APD with NDDs
Oscillatory network regulation (gamma/ASSR) [11,12]	Supports neural synchrony for auditory temporal encoding	Reduced 40 Hz ASSR power; altered gamma activity in ASD and related conditions	Candidate electrophysiological marker of central auditory dysfunction

**Table 4:** Atypical development or function of inhibitory circuits can directly impair auditory perception. Key neurophysiological and neurochemical markers linked to E–I imbalance in auditory processing disorders.

Method	Marker	Neurotransmitter Link	Clinical Relevance
Magnetic resonance spectroscopy (MRS) [20,46]	GABA, glutamate, Glx concentrations	Reflects E/I balance in auditory cortex	Candidate biomarker for APD, tinnitus, ASD
Auditory steady-state response (ASSR) [11,12]	Gamma-band synchrony (≈40 Hz)	Strong dependence on GABAergic inhibition	Marker of temporal coding and sensory gating deficits
EEG resting-state oscillations [47]	Theta increase, beta reduction	Suggest altered inhibitory control	Associated with listening difficulties and attention deficits
Long-latency auditory evoked responses (LLAER) [48]	N1/N2 amplitude and latency changes	Reflect cortical auditory processing efficiency	Possible diagnostic marker for CAPD
Behavioral auditory tests [3,8]	Speech-in-noise, temporal processing	Indirect index of E/I balance	Essential for clinical diagnosis but influenced by cognition

**Table 5:** The main functional roles and auditory-related implications of dopamine, serotonin, and norepinephrine.

Neurotransmitter	Core Neurobiological Functions	Role in Auditory Processing	Evidence in NDD/APD	Potential Clinical Implications
Dopamine [18,39,56,57]	Reward processing, motor control, motivation, executive regulation	Modulates neuronal responses in the inferior colliculus; influences attentional filtering and salience attribution of auditory stimuli; regulates E/I balance via interneurons	Altered dopaminergic signaling reported in ADHD, ASD, Parkinson’s disease; associated with speech perception deficits and attentional listening difficulties	May contribute to listening difficulties linked to attentional control; potential target mainly when auditory deficits co-occur with attentional dysfunction
Serotonin [17,19]	Mood regulation, sensory modulation, cognitive flexibility, behavioral adaptation	Influences temporal auditory processing, sensory gating, multisensory integration, and neural gain control; modulates phase-locking to acoustic stimuli	Hyperserotoninemia reported in ASD; serotonergic modulation improves auditory temporal processing and central auditory responses	Potential therapeutic target for sensory filtering and temporal processing deficits; involvement in multisensory integration abnormalities
Norepinephrine [40,41]	Arousal, vigilance, attentional regulation, adaptive behavioral states	Regulates auditory attention, signal-to-noise extraction, and sensory gain control; supports sustained listening in complex acoustic environments	Noradrenergic dysregulation implicated in ADHD and age-related cognitive decline; associated with auditory attention deficits	Pharmacological enhancement (e.g., atomoxetine) may improve attention-dependent auditory processing and executive listening functions

## Data Availability

This is a review and all the articles cited are in the references section.
